# Mesenchymal Stem Cell-Derived Exosomes as a Novel Strategy for the Treatment of Intervertebral Disc Degeneration

**DOI:** 10.3389/fcell.2021.770510

**Published:** 2022-01-24

**Authors:** Lin Lu, Aoshuang Xu, Fei Gao, Chenjun Tian, Honglin Wang, Jiayao Zhang, Yi Xie, Pengran Liu, Songxiang Liu, Cao Yang, Zhewei Ye, Xinghuo Wu

**Affiliations:** ^1^ Department of Orthopaedics Surgery, Union Hospital, Tongji Medical College, Huazhong University of Science and Technology, Wuhan, China; ^2^ Institute of Hematology, Union Hospital, Tongji Medical College, Huazhong University of Science and Technology, Wuhan, China; ^3^ The First Clinical Medical College of Lanzhou University, Lanzhou, China; ^4^ The First Hospital of Lanzhou University, Lanzhou, China

**Keywords:** exosomes, extracellular vesicles (EVs), mesenchymal stem cell (MSC), IVD degeneration (IVDD), microRNA

## Abstract

Intervertebral disc degeneration (IVDD) has been reported to be the most prevalent contributor to low back pain, posing a significant strain on the healthcare systems on a global scale. Currently, there are no approved therapies available for the prevention of the progressive degeneration of intervertebral disc (IVD); however, emerging regenerative strategies that aim to restore the normal structure of the disc have been fundamentally promising. In the last decade, mesenchymal stem cells (MSCs) have received a significant deal of interest for the treatment of IVDD due to their differentiation potential, immunoregulatory capabilities, and capability to be cultured and regulated in a favorable environment. Recent investigations show that the pleiotropic impacts of MSCs are regulated by the production of soluble paracrine factors. Exosomes play an important role in regulating such effects. In this review, we have summarized the current treatments for disc degenerative diseases and their limitations and highlighted the therapeutic role and its underlying mechanism of MSC-derived exosomes in IVDD, as well as the possible future developments for exosomes.

## 1 Introduction

Low back pain (LBP) is a widespread health concern in modern society, resulting in high socioeconomic consequences (Katz, 2006; Kague et al., 2021). Epidemiological statistics show that approximately 80% of adults experience lower back pain at least once in their lifetime (Park et al., 2021); LBP is considered to be one of the most important reasons that limit the working capacity of people younger than 45 years (Deyo and Weinstein, 2001; Balague et al., 2012). Annually, the direct cost of treating LBP is approximately US$ 30 billion and the indirect socio-economic losses account for approximately US$ 100 billion in the United States (Binch et al., 2021). The incidence and prevalence of LBP in China have increased annually, and it has become one of the major diseases that affect both public health and people’s quality of life (Yao et al., 2011; Wu et al., 2019). Previous studies (Liao et al., 2019a; Au et al., 2020; Binch et al., 2021; Croft et al., 2021) showed that intervertebral disc (IVD) degeneration (IVDD) is the most prevalent contributor to LBP at present. Although the etiology of IVDD remains poorly understood, some influencing factors include metabolic disorders, aging, mechanical loading, trauma, infection, nutrition, and genetic predisposition (Deyo and Weinstein, 2001; Cherif et al., 2020). To date, the clinical treatment for LBP mainly includes bed rest, administration of non-steroid anti-inflammatory drugs (NSAIDs) and analgesics, lumbar discectomy, and interbody fusion (Binch et al., 2021). However, these approaches only focus on temporarily alleviating the symptoms rather than targeting the pathogenesis, and as such the progression of IVDD is neither delayed nor reversed (Hu et al., 2020; DiStefano et al., 2021). Therefore, novel treatment modalities are needed in IVDD therapy. In recent years, indeed, emerging strategies for the treatment of IVDD has gained the attention of researchers; MSC-based therapies and exosomes from MSCs show promising effects and have potential clinical applicability (Melrose, 2016; Richardson et al., 2016; Vadala et al., 2016; Loibl et al., 2019; Croft et al., 2021). The efficacy of MSCs in IVDD repair has been demonstrated in preclinical studies (Orozco et al., 2011; Peroglio et al., 2013; Zhang et al., 2021), and more recently in clinical trials (Elabd et al., 2016; Centeno et al., 2017; Pettine et al., 2017). Originally, it was thought that the injected MSCs could phenotypically differentiate into nucleus pulposus-like cells to replace the damaged tissue (Risbud et al., 2004a; Risbud et al., 2004b; Steck et al., 2005). However, this hypothesis was later challenged by the findings from numerous studies in the last decade. There exists a consensus that MSCs exert their therapeutic functions *in vivo* mostly via the paracrine process, such as the release of signaling molecules including growth factors, cytokines, and extracellular vesicles (Fayazi et al., 2021; Mohd Noor et al., 2021). Among them, exosomes, as the main transmission medium for intercellular communication, participate in a variety of pathological and physiological functions by means of influencing the gene expression, migration, proliferation, apoptosis, and metabolism in the recipient cells (Pourakbari et al., 2019; Shi et al., 2021). Recent years have witnessed an increase in the amount of research being carried out on the therapeutic value of exosomes for the treatment of IVDD. In view of this, we have concentrated our attention on IVDD, MSCs, and exosomes in the present research. Specifically, we focused on the potential mechanisms of MSC-derived exosomes for IVDD treatment.

## 2 Pathology of IVDD

**FIGURE 1:**
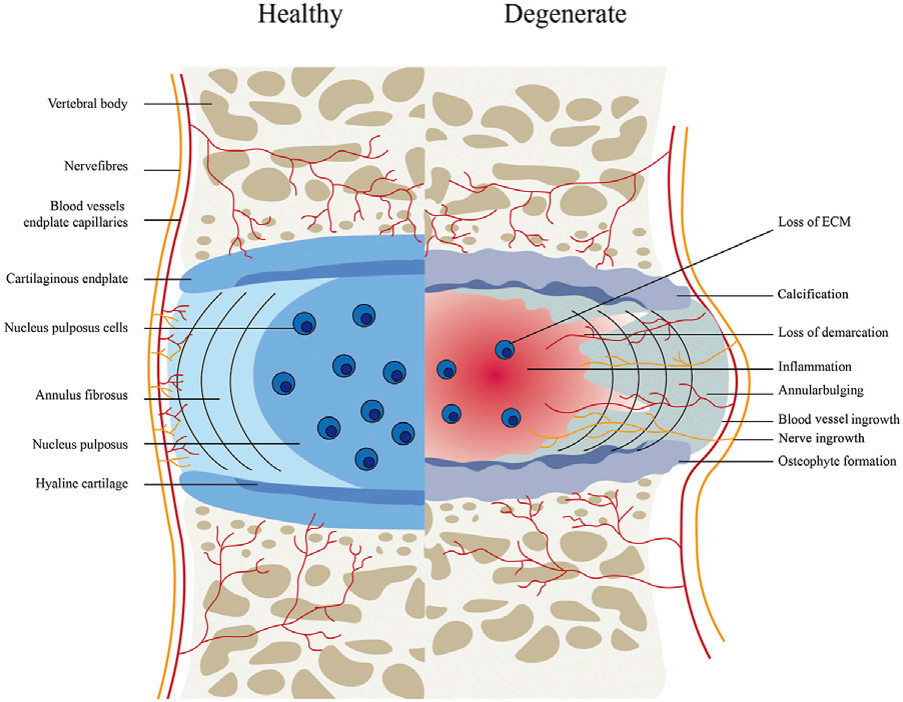
The intervertebral disc in healthy and pathological tissues.

The intervertebral consists of an outer annulus fibrosus (AF) and center gel-like nucleus pulposus (NP), as well as 2 cartilaginous endplates (Zhang et al., 2019; Binch et al., 2021). In the human body, the IVD is known to be the largest avascular tissue (Liao et al., 2019b), the IVD has a very limited intrinsic healing potential. Several pathophysiological processes of IVDD include decreased proliferation and increased apoptosis of nucleus pulposus cells, growth of nerves and blood vessels into intervertebral disc tissue, metabolic disorders (decreased synthesis and increased degradation) of extracellular matrix, aging of annulus fibrosus, and calcification of cartilage endplate (Balague et al., 2012). These eventually result in the clinical symptoms due to the inability to maintain the normal hydrophilic characteristics of the intervertebral disc, a large loss of water content, partial atrophy of nucleus pulposus, the disappearance of the normal borderline between annulus fibrosus and nucleus pulposus, and loss of intervertebral height (Deyo and Weinstein, 2001) (Figure 1).

## 3 Current Treatments for Intervertebral Disc Degeneration Diseases

### 3.1 Conservative and Surgical Treatments

At present, IVDD therapy generally includes conservative symptomatic and surgical treatments (Park et al., 2021). Conservative treatment usually exhibits good results for LBP (van Middelkoop et al., 2011; Quentin et al., 2021). It primarily includes oral drugs (including opioids, non-steroidal anti-inflammatory drugs (NSAIDs), skeletal muscle relaxants, acetaminophen), epidural steroid injections, and physical therapy. Surgical treatment tends to be restricted to people with severe or worsening symptoms, often after the conservative treatment has failed (Deyo and Weinstein, 2001; Binch et al., 2021). It includes interventional surgery (ozone radiofrequency ablation, etc.) and conventional surgery (discectomy, fusion, etc.) (Acosta et al., 2005; Binch et al., 2021). However, whether conservative treatment or surgical treatment are mainly targeted to the alleviation of pain symptoms rather than the underlying pathogenesis or reversing the process of IVDD and, although many patients respond in the short term, they generally have limited or no long-lasting clinical benefit. There are now a variety of biological and regeneration therapies aiming at targeting the illness at the cellular and molecular levels and reestablishing IVD functioning. As a result, these therapies have the potential to provide better clinical results.

### 3.2 Biologic and Small-Molecule Therapies

Since the exact mechanism of IVDD has not been completely understood, conservative therapies seem to be mostly inefficacious, and contemporary surgical interventions concentrate predominantly on removing pathological disc tissue and fusing the spine1. Various novel biologic therapies for IVDD degeneration have been evolved, including growth factor injection, platelet-rich plasma, gene therapy, cell-based therapy, and so on. Studies on small-molecule therapies have also been conducted.

#### 3.2.1 Growth Factor-Based Therapy

The homeostasis of IVDD is regulated by actively maintaining the balance between catabolism and anabolism of IVD cells. Several growth factors have been reported to maintain this balance (Kennon et al., 2018). Han et al. (2015) have suggested that the direct injection of growth factors was among the most promising biological treatment modalities for the regeneration or repair of an IVD that has suffered from deterioration. Accumulating evidence implies that growth factors including growth and differentiation factor-5 (GDF-5), TGF-β, BMP-2, OP-1, basic fibroblast growth factor (bFGF/FGF2), epidermal growth factor (EGF), and IGF-1 in some models could promote cell proliferation and matrix synthesis (Imai et al., 2007; Ellman et al., 2008; Masuda, 2008; Cho et al., 2013; Travascio et al., 2014). It is worth noting that the response of different growth factors to different matrix structures and cellularity of the deteriorating disk is heterogeneous. Exogenous GDF-5, BMP-2, and TGF-βrepaired inner annular chondrocytes in degraded IVDs by promoting synthesis of NP and inner annulus, while IGF-1/b-FGF had little effect on these cells (Imai et al., 2007; Masuda, 2008; Cho et al., 2013; Travascio et al., 2014). Proteolytic enzymes such as ADAMTS and MMPs, as well as pro-inflammatory cytokines, act as an important role in the ECM degradation of NP. So, the injection of specific inhibitors or antagonists of these enzymes into the IVD is considered as a viable method to inhibit further degradation of ECM which was caused by the target protein hydrolases (Vo et al., 2013; Han et al., 2015). However, owing to the biological factors having short half-lives, the extremely low number of cells that are viable in terms of the capacity to be activated in IVDs that have already deteriorated IVDs, the limited effect of a single injection of growth factors, and the additional trauma and inflammatory reactions of repeated injections, all these factors make it difficult to apply growth factor-based therapy to clinical treatment (Mackiewicz et al., 2009; Kepler et al., 2013).

#### 3.2.2 Platelet-Rich Plasma Therapy

Platelet-rich plasma (PRP) is known as an autologous blood concentrate of platelets. Once activated, it secretes a variety of chemokines, cytokines, growth factors, and other functional molecules (Wang et al., 2015). As a cocktail of multiple bioactive factors, PRP is widely clinically used as a therapeutic agent for tissue repair and regeneration. Several *in vivo* and *in vitro* investigations have shown the utility of PRP for IVD repair and regeneration (Obata et al., 2012; Wang et al., 2013; Pirvu et al., 2014). In addition as a therapeutic strategy, PRP has the merits of being low-cost, ease of preparation, ease of application, and autologous sources without rejection reaction (Chang and Park, 2021). Nevertheless, in view of the possible negative consequences of ossification, inflammation, and vascularization, more research on standardized formulations is of great necessity to validate the effectiveness and safety of PRP.

#### 3.2.3 Gene Therapy

When compared to single injections of bio factors, gene therapy techniques are expected to possess a longer duration of action. Gene therapy is the process of delivering one or even more desirable genes into target cells with the aid of a vector (primarily a viral or non-viral vector) for the purpose of modifying the functioning state of targeted cells by releasing the product of the donor gene (Sakai and Grad, 2015). A large number of research reports have established strategies for delivering the desirable gene into IVD cells (Zhang et al., 2009; Liang et al., 2010; Ren et al., 2013). IVD regeneration has been investigated thoroughly using desired genetic encoding for anabolic factors (LMP-1, GDF-5, and BMPs), the transcription factor Sox-9 (Paul et al., 2003), anti-inflammatory agents (IL1ra) (Le Maitre et al., 2007), or metalloproteinase inhibitors (TIMP-1) (Wallach et al., 2003). Though gene therapy has shown promise, safety issues associated with immunogenicity, insertional mutations, and unregulated gene expression, as well as the assessment of long-term efficacy, must also be determined and addressed seriously. Furthermore, gene therapy is not a cost-effective solution because of the significant expense involved in the preparation of the vectors for gene delivery.

#### 3.3.4. Small Molecules Therapy

Small molecular are defined as therapeutics with molecular weights of 600D or less, that can specifically bind to certain biological intracellular molecules and play a regulating role in particular biological processes (O'Shea et al., 2013). Unlike traditional drugs, small molecules are usually inhibitors of the relevant signaling pathway, which provides a promising target in the development of IVDD (Chen et al., 2017; Binch et al., 2021). Several studies have shown that small-molecule exhibited beneficial effects mainly through attenuation inflammatory signaling, apoptosis, catabolism, oxidative stress, and activation of anabolism by regulating a variety of signaling pathways (Colombier et al., 2014; Sakai and Grad, 2015; Chen et al., 2016; Li et al., 2017a; Pan et al., 2018; Zhang et al., 2018; Lin et al., 2019). Above, small molecular therapy is a positive alternative therapy for IVDD. Several of these small molecules have been shown to be effective in preventing disc cell deterioration. Furthermore, they are also crucial components in the regeneration of injured IVD in view of the fact that they regulate enzymes that are involved in the metabolism process (Kamali et al., 2021). Additionally, in contrast with cell- or growth factor-based treatment, small molecules are rarely biodegraded *in vivo* and are usually seen as a more cost-effective treatment option (Sun et al., 2019). However, small-molecule drugs still have relatively few clinical applications in IVDD. Currently, they do not show significantly better than NSAIDs based on existing clinical evidence (Daily et al., 2016; Chen et al., 2017; Grothe et al., 2017). The possible reasons are as listed: Firstly, small molecules always have weak specificity, which may affect other tissues resulting in unexpected side effects when systemically administered. Secondly, single-target strategies have not always brought optimistic results, due to complex diseases often needing to be tracked at various levels by modulating several targets simultaneously. The third reason is the limited number of clinical trials that have been conducted in this field, inadequate *in vivo* data as a consequence of a scarcity of comparable animal models, and the absence of patient stratification techniques to determine who would gain considerable benefit from a small molecule-based therapy.

Next-generation treatment strategies are based on regenerative medicine (Fernandez-Francos et al., 2021). The aim is to develop therapies that prevent LBP by delaying the IVD deterioration and promoting its repair. These strategies can be roughly split into two groups: cell-based and cell-free therapies (DiStefano et al., 2021); among the cell-free systems, exosomes have received a great deal of interest as a potentially effective therapeutic approach for IVDD in recent years.

## 4 Mesenchymal Stem Cell-Based Therapy in IVDD Treatment

MSCs are pluripotent non-hematopoietic stem cells that may be obtained from a wide range of sources, such as the umbilical cord blood, urine, adipose tissue, liver, kidney, bone marrow, placenta, and so on (Meng et al., 2013; Quang et al., 2018). A minimum of three conditions must be met by MSCs in accordance with the report released by the International Society for Cellular Therapy (ISCT) (Dominici et al., 2006). In addition, when developed *in vitro*, MSCs are considered to be plastic-adherent. Moreover, MSCs should express the surface markers CD105, CD90, and CD73, while expressing none of the hematopoietic markers CD34, CD45, CD11b or CD14, CD19 or CD79α and HLA-DR. Furthermore, when grown under particular circumstances, MSC should be differentiated into chondroblasts, adipocytes, and osteoblasts. Different MSCs have emerged as trendsetters in the field of cell therapy research due to their ease of isolation and specific biological functions (Wang X. et al., 2019). On the one hand, because of their pluripotency, MSCs were believed to replicate and differentiate into specialized cells to replace the damaged tissue (Richardson et al., 2016; Fernandez-Francos et al., 2021); MSCs, on the other hand, may produce bioactive molecules in response to their contact with the host niche including platelet-derived growth factor (PDGF), matrix metalloproteinase-9, matrix metalloproteinase-2 (MMP-2), prostaglandin E2 (PGE2), interleukin-6 (IL-6), and, insulin-like growth factor-1 (IGF-1), and ultimately have the functions of anti-apoptosis, immunomodulatory, anti-inflammatory, angiogenic and anti-fibrosis (Ha et al., 2020; Sun S.-J. et al., 2021; Shi et al., 2021). As a consequence, the utility of MSC-based therapeutics has been brought to the forefront of research. Actually, mesenchymal stem cells (MSCs) have shown safety as well as therapeutic significance in the field of regenerative medicine (Mocchi et al., 2020). Currently, about 1,000 clinical trials worldwide have been reported to use MSCs to treat various diseases (Pittenger et al., 2019; Kabat et al., 2020), including inflammatory and fibrotic diseases (Ryu et al., 2020); liver disease (Zhu et al., 2021), bone and joint diseases (Li N. et al., 2020), inflammatory bowel diseases (Zhang X. et al., 2020), diabetes (Kuang et al., 2021), cardiovascular diseases (Chen et al., 2021), neurodegenerative diseases (Fayazi et al., 2021), spinal cord injuries (Huang et al., 2021) and so on. Likewise, the emergence of cell-based therapies has also paved the way for utilizing MSCs in IVDD treatment. A large number of preclinical studies have established the effectiveness of MSCs in the treatment of IVDD (Vadala et al., 2016; Sinkemani et al., 2020). On the one hand, under appropriate culture conditions, mesenchymal stem cells may differentiate into NP-like cells. For example, Stoyanov et al. (2011) examined if it was feasible to guide bone marrow-derived MSCs to take on the phenotype of natural killer cells (NPs). They discovered that hypoxia, growth/differentiation factor 5 (GDF5), and induction of markers associated with NP-cell-like phenotype by NPC co-culture have been shown to have an effect on the expression profiles of bone marrow-derived MSCs in humans. More recently, a similar finding was reported by Clarke et al. (2014). Other growth factors, including transforming growth factor-beta 3 (TGF-β3) and especially growth/differentiation factor 6 (GDF6), in addition to GDF5, were shown to significantly elevate the NP marker genes expressions in MSCs. Moreover, in an animal experiment *in vivo*, Sakai et al. (2005) transplanted autologous bone marrow MSCs labeled with green fluorescent protein into a rabbit model of the IVDD. Two weeks after transplantation, a large number of cells expressing green fluorescent protein were found in the transplantation space. Compared with the non-transplantation group, the expression of type II collagen and proteoglycan in the intervertebral disc of the transplantation group was substantially elevated, which indicated that the transplanted MSC could differentiate into the nucleus pulposus cells in the hypoxic environment of the IVD.

On the other hand, MSCs are reported to produce a wide range of trophic factors to rescue and activate nucleus pulposus cells isolated from degenerative intervertebral discs by enhancing ECM synthesis, down-regulating pro-inflammatory cytokines, and up-regulating NP marker genes (including SOX9, type II collagen, and proteoglycans) within the diseased disc (Barakat et al., 2019). In many preclinical studies promoting IVD regeneration, the role of this MSC paracrine has been proved (Qi et al., 2019; Zeng et al., 2020). A study (Qi et al., 2019) that induced nucleus pulposus mesenchymal stem cells (NPMSCs) apoptosis through high glucose (HG) treatment found that MSC conditioned medium (MSC-CM) has resumed the collagen II and aggrecan expression in NPMSCs, thereby alleviating extracellular matrix degradation. Another study (Zeng et al., 2020) also demonstrated that CM derived from umbilical cord-derived MSCs (UCMSCs) might enhance the regeneration ability of nucleus pulpo-sus-derived progenitor/stem cells from degenerated disc (D-NPSCs) by enhancing their proliferative and stemness abilities, indicating the prospects of treatment approach to reinvigorate IVD progenitor/stem cells via paracrine signaling, which could be achieved with the aid of viable factors generated from MSCs.

The utility of cell-based MSC treatment, on the other hand, has certain limitations. First, although results of cell transplantation (through injection) are preliminary for the treatment of intervertebral disc degeneration, most studies showed that the transplanted cells could not adapt to intra-disc ischemia and hypoxia and thus could not survive (Binch et al., 2021). This greatly limits the therapeutic effect of stem cells. Second, MSC-based therapies may lead to the risk of granulocytosis, graft rejection, ectopic bone formation, microvascular embolism, and tumorigenicity (Ryu et al., 2020; Sun S.-J. et al., 2021). Third, the standardized culture procedures, the timings, the methods of treatment, and the selection of donor cells need to be studied further (Binch et al., 2021). Therefore, an effective treatment strategy should not include the direct participation of stem cells. Indeed, accumulating evidence showed that the efficacy of cell-based therapies is mainly attributed to paracrine signaling (Ha et al., 2020; Ratajczak and Ratajczak, 2020; Bao and He, 2021; DiStefano et al., 2021). Novel therapeutic methods are developed as a result of this discovery, including the creation of cell-free techniques on the basis of the utilization of secretome fractions such as vesicular and soluble fractions as a possibly more attractive option to cell treatment. The identification and application of soluble vesicles in the secretome as a substitute for cell-free therapy is a hot spot of current research. As most cell types, including MSCs, can produce exosomes with comparable therapeutic effects, exosomes containing various paracrine mediators are a potential and attractive cell-free therapeutic strategy for IVDD.

## 5 MSC-Derived Exosomes

### 5.1 Exosome Biogenesis

**FIGURE 2:**
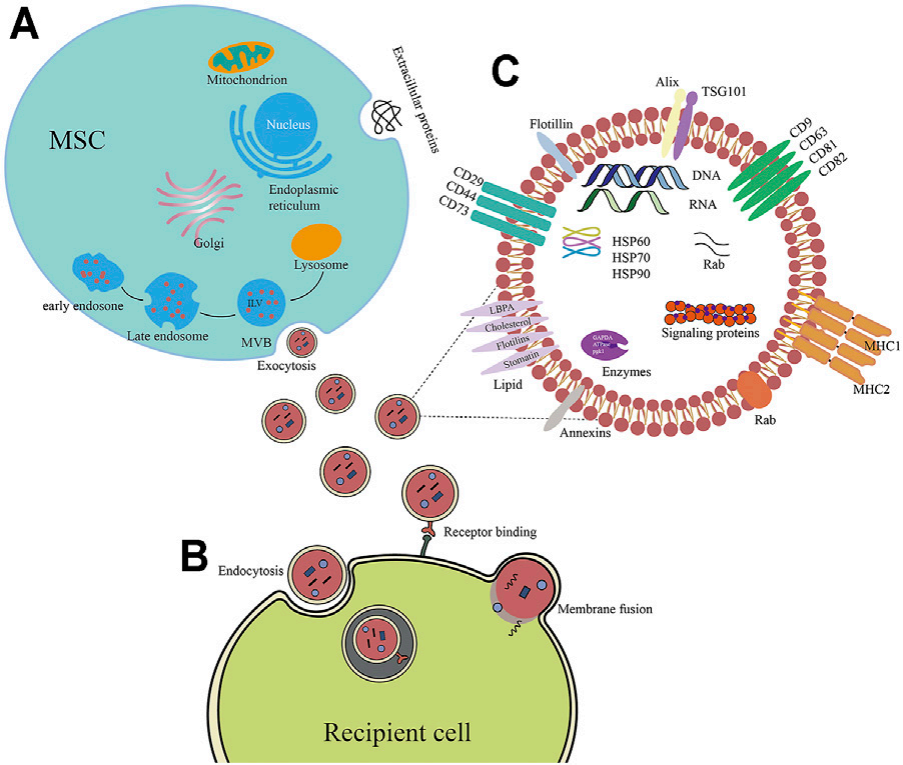
**(A)** The molecular composition of exosomes generated from MSCs. Exosomes generated from MSC contain adhesion molecules (CD73, CD44, and CD29), tetraspanins (CD9, CD63, and CD81), multivesicular body biogenesis-related proteins (Tsg101, Alix), and heat shock proteins. In addition to proteins, exosomes also comprise enzymes, lipids, and nucleic acids (DNA, miRNA, microRNA, and mRNA). **(B)** Biogenesis of exosomes produced from MSCs. As exosomes, these vesicles of endocytic origin are generated when the multivesicular body (MVB) membrane is subjected to inward budding, resulting in the formation of exosomes. ILV is found in MVBs. The production of exosomes occurs as a consequence of the fusion of MVBs with their plasma membrane. **(C)** Exosomes may infiltrate target cells by a variety of pathways, including membrane fusion, binding receptors, and endocytosis.

Exosomes, a subset of extracellular vesicle (EVs), are phospholipid vesicles that are membrane-bound and released by nearly all tissues, cells, and body fluids (plasma, urine, saliva, amniotic fluid, gastrointestinal secretions, semen, synovial fluid, and breast milk) (Pegtel and Gould, 2019). EVs are classified into three categories as follows: exosomes (30–100 nm), microparticles (100–1,000 nm), and apoptotic bodies (1,000–5000 nm) (Malekpour et al., 2021) (Table 1). In addition to the differences in their size, the biogenesis of exosomes is a hallmark feature that distinguishes them from other EVs. Unlike microvesicles which are generated directly from the plasma membrane and apoptotic bodies that are generated in the process of apoptosis, the exosomes have endosomal origins (Birtwistle et al., 2021). Exosomes are derived from an endosomal compartment, then released into the extracellular environment upon fusion of multivesicular bodies (MVBs) comprising of intraluminal vesicles (ILVs) with the plasma membrane. (Figure 2). The endosomal sorting complexes required for transport (ESCRT) located on MVBs regulate exosomes loading and biogenesis (Birtwistle et al., 2021). The ESCRT is a family of proteins that associate in successive complexes (ESCRT-0, -I, -II, and -III) at the membranes of MVBs for the purpose of regulating the production of ILVs as well as their cargo (Gupta and Pulliam, 2014). Many associated proteins (such as Tsg101, Alix, VPS4) also participate in these processes (Birtwistle et al., 2021). Tsg101 (typical tumor susceptibility gene 101 protein) is a component of the cellular ESCRT-I complex, whereas Alix (encoded by PDCD6IP) is an auxiliary protein of the ESCRT-III complex (Wang B. et al., 2019). Furthermore, exosome biogenesis may be regulated by ESCRT-independent machinery, which is reliant on Hsp70 binding phospholipids to construct MVBs (DiStefano et al., 2021). Moreover, the biogenesis, as well as the release of exosomes generated by MSC, are both dependent on external cues to function normally. Moreover, under various stimuli, different contents and compositions of exosomes secreted from the same parental cells were observed (Stoyanov et al., 2011; Babaei and Rezaie, 2021). Upon being produced from the cell, exosomes have been found to be either guided to and taken up by recipient cells that are present in the microenvironment or are transported to other regions by the body’s circulatory system. The exosomes are mostly taken up by the recipient cells via three routes: 1) ligand-receptor interaction; 2) endocytosis of target cells; 3) direct membrane fusion (Sun S.-J. et al., 2021; Fayazi et al., 2021; Liu et al., 2021) (Figure 2). The release of exosome contents into the cytoplasmic space of recipient cells occurs after the exosomes merge with recipient cells, facilitating the horizontal transmission of their cargo.

**TABLE 1:** Summary of details for exosomes, microvesicles, and apoptotic bodies.

Different types of EVs			
Characteristics	Exosomes	Microvesicles	Apoptotic bodies
Size (nm)	50–150 nm	100∼1,000 nm	1,000∼5000 nm
Morphology	Cup-shaped	Heterogenous	Heterogenous
Origin	Endosomal compartment of cells; Multivesicular body (MVB)	Plasma membrane	Apoptotic cell membrance
Mechanism of discharge	Exocytosis of MVB	Budding off the plasma membrane	Outward blebbing of the cell membrane
Biomarkers	CD63, CD9, CD81, aliX, TSG101, tetraspanins, HSP70, etc.	CD40 ligand, selectins, integrins	Histones, Annexin V
Cargos	mRNA, microRNA, lncRNA, circRNA, DNA lipid, protein, etc.	mRNA, microRNA, other non-coding RNA, protein, etc.	Nuclear fractions, cell organelles, etc.

ALIX, apoptosis-linked gene 2-interacting protein X; CD, cluster of differentiation; HSP70, 70 kilodalton heat shock proteins; TSG101, tumor susceptibility gene 101 protein.

### 5.2 MSC-Derived Exosomes Components

Exosomes’ molecular structure is highly variable, depending on the parent cell type, epigenetic modifications, and numerous pathophysiological environmental factors (DiStefano et al., 2021). The exosomal components mainly include nucleic acids, proteins, and lipids and these determine the biological functions of the exosomes. In recent years, with the rapid development in biotechnology including transcriptomics, proteomics, lipidomics, and bioinformatics, the compositions of exosomes have been well studied, which provide a theoretical basis for exocrine therapy of diseases (Yu et al., 2014). The exosomal lipids mainly include phosphatidylserine, phosphatidic acid, cholesterol, sphingomyelin, arachidonic acid, prostaglandins, and leukotrienes; these not only participate in the formation of exosomes but also performs an instrumental function in the maintenance of their biological stability (Pegtel and Gould, 2019). Exosomes are also abundant in a variety of multifunctional proteins, including those involved in exosome synthesis (for example, the syntenin, ALIX, TSG101, and ESCRT complex), as well as membrane transporter and fusion proteins (for example, annexins, heat shock protein, and, Rab GTPases) (Xunian and Kalluri, 2020). In addition to proteins and lipids, various nucleic acids such as genomic DNA, cDNA, mitochondrial DNA (mtDNA), long noncoding RNAs (lncRNAs), circularRNAs, microRNAs (miRNAs), and mRNAs are excessively enriched in exosomes (Yu et al., 2014; Nakamura, 2019). These molecules have a role in the epigenetic remodeling of cells and the modulation of biological functions. Nevertheless, the cargo of each exosome colony differs significantly depending on the tissue and cell types of the secretory population (Ibrahim and Marban, 2016).

MSCs are known to produce more exosomes than do other types of primary cells (Yu et al., 2014). Proteomic analyses show that a total of 1,927 different proteins in MSC exosomes have multifaceted functions necessary for their functional and biogenesis characteristics (Li et al., 2020b). Not only do MSC-derived exosomes express ubiquitous exosome surface proteins including tetraspanins (CD81, CD63, and CD9), Alix, and Tsg101, as well as heat shock proteins (HSP90, HSP70, and HSP60), but also the MSC membrane proteins for several adhesion molecules such as CD73, CD44, and CD29. (Schorey and Bhatnagar, 2008; Colombo et al., 2014) (Figure 2). Like general exosomes, diverse types of nucleic acids including lncRNAs, miRNAs, and mRNA are enriched in MSC exosomes (Vlassov et al., 2012). They perform an integral function in changing the fate of recipient cells. Through the mechanism of exosome-cell contact, exosomal RNAs may be translated inside recipient cells, resulting in gene suppression and post-transcriptional modulation (Wu et al., 2020). Among them, micro-RNAs (miRNAs) have constantly garnered greater interest (Li et al., 2019; Zhang C. et al., 2020). miRNAs are thought to be a form of non-coding RNA which are approximately 22 nucleotides in length (Xie et al., 2020). They modulate post-transcriptional gene expression as they bind to the 30 UTR of the target mRNAs (Ma et al., 2020). miRs are among the major MSC exosomal effector molecules, which can participate in multiple biological processes including cell differentiation, apoptosis, angiogenesis, and inflammatory pathways. For example, tumor growth inhibitor miRs (miR-122, miR-125b, miR-24, miR-31, miR-223, miR-451, miR-214, and miR-23b) (Hassanzadeh et al., 2021) and inflammation modulatory miRs (miR-146 and miR-155) (Hulsmans and Holvoet, 2013) have been reported in MSC-derived exosomes. Notably, a single miR may control numerous messenger RNAs and a single messenger RNA could also be modulated by many miRs (Jin and Lee, 2015; Kong et al., 2015). These complex regulatory networks confer on MSC exosomes the potential to produce multiple functional effects, which in turn mediate various physiological and pathological processes.

## 6 Methods for Exosome Isolation and Identification

### 6.1 Isolation of Exosomes

There are several isolation techniques including centrifugation, ultrafiltration, size exclusion chromatography, polymer precipitation, immune-affinity capture, and microfluidics.

Centrifugation could either be ultracentrifugation or sucrose density gradient centrifugation. Ultracentrifugation is most commonly used and is known as the “gold standard” for separating exosomes (Ailuno et al., 2020). Ultracentrifugation is based on the differential sedimentation rate of proteins, vesicles, cell debris, and cells in uniform suspensions. By progressively elevating the centrifugal force and duration, cells and bigger particles can be eliminated. The exosomes are separated at ≥100,000 g/min and >2 h centrifugation (Gupta et al., 2018). As the density of exosomes is approximately 1.13∼ 1.19 g/ml (Raposo et al., 1996; Thery et al., 2006; Tauro et al., 2012), the density gradient centrifugation can be used for their separation. This method combines ultracentrifugation with a sucrose cushion or sucrose density gradient, and is based on the principle of separation and concentration according to gradient density after the removal of non-vesicular substances; exosomes with higher purity can be obtained using this method (Tauro et al., 2012). However, this method is time-consuming and has a lower yield. The quantity and quality of exosomes obtained are susceptible to uncertainties due to external factors. Further, it is difficult to separate other impurities of similar density, such as vesicles and proteins, from the obtained exosomes (Wang et al., 2021).

Size exclusion chromatography (SEC) is a chromatographic technique that separates small-molecule–protein complexes based on their size. (Veyel et al., 2018). The technique requires a chromatographic column made of porous polymer. First, the process involves removing cells as well as bigger particles via centrifugation at a low speed, subsequently filtering twice with a filter, and final purification by SEC (Sokolova et al., 2011). The most dazzling advantage of this method is that it requires only low centrifugal force, and hence avoids the demolition of the vesicle structure to a greater extent. However, the purity of the produced exosomes is lesser. The chromatogram column is easy to block and the adhesion leads to the loss of exosomes (Tauro et al., 2012; Xu et al., 2019).

Ultrafiltration (UF) is another technique of separation based on the size of exosomes (Sidhom et al., 2020). According to the diameter of exosomes, an ultrafiltration membrane that corresponds to the closest molecular weight is used to separate exosomes by centrifugation or pressurization (Li et al., 2017b). It is simple and rapid, but it has an obvious drawback that it is difficult to separate exosomes from other extracellular vesicles with similar diameters (Yu et al., 2018). The adhesion to the ultrafiltration membrane reduces the yield of exosomes, and the external force applied in the process of ultrafiltration may also cause the deformation or rupture of exosomes.

Isolation by immune-affinity chromatography is based on the interaction between antigens and antibodies with high specific affinity (Schiel and Hage, 2009). It is mainly used for the separation and purification of biological macromolecules. Certain antibodies against specific exosome surface indicators, including tetraspanins and TSG101 proteins, are generally utilized for exosome extraction and purification (Mondal and Whiteside, 2021).

Polymer precipitation is a process in which exosomes are precipitated from body fluids or cell cultures by altering their solubility or dispersion (Wei et al., 2021). It is usually used to precipitate exosomes from samples using polyethylene glycol or agglutinin. Currently, commercial kits such as ExoQuick and Exoquick-Tc kits from System Biosciences Company are available for extracting exosomes using polyethylene glycol (PEG) solutions. It is simple to use and does not require any special equipment; however, it is expensive. The reaction with body fluids or cell culture medium is incubated overnight at 4 °C and then centrifuged at a low speed.

Microfluidics is a new technology that uses micro-and nano-sized tubes to process and manipulate fluids (Chen et al., 2020). It can be divided into three categories: immune affinity, screening, and porous structure capture (Liga et al., 2015). Compared to the other techniques, this method is portable, fast, cost-effective, and automated, with a higher recovery rate. However, as it is a new emerging technology, it lacks standardization and large-scale sample data. Hence this method needs to be verified in future studies.

### 6.2 Identification of Exosomes

Currently, exosomal identification generally relies on the morphology, size, and their marker proteins using various methods including western blot analysis (WB), flow cytometry (FCM), nanoparticle tracking analysis (NTA), atomic force microscopy (AFM), scanning electron microscope (SEM), and transmission electron microscope (TEM) (Sokolova et al., 2011; Yang et al., 2019). The size and morphology of exosomes are usually observed using TEM, SEM, and AFM; among these TEM is most commonly used (Liu et al., 2018). NTA is used for the purpose of examining the exosome concentration and diameter. FCM and WB may detect exosomal surface marker proteins, including CD82, CD81, CD63, and CD9 (Yaghoubi et al., 2019). Usually, exosome identification involves a combination of two or more of these methods for a comprehensive assessment.

## 7 The Therapeutic Promise of Exosomes Generated From MSCs in a Variety of Tissues

Exosomes have been shown to have favorable effects in a significant number of experiments conducted over the last decade. Compared to the MSCs, MSC-derived exosomes not only play an indispensable role in tissue repair and regeneration but also have a more lasting, potent, and “easier-to-preserve” role (Mohd Noor et al., 2021). Owing to their small size, MSC-derived exosomes have greater stability and mitigate the risks associated with the administration of live cells, such as the possibility of microvascular blockage throughout the body. In addition, exosomes as small transporters can also easily get through the blood-brain barrier, while MSCs cannot.

In fact, many pre-clinical trials have illustrated the importance of exosomes derived from MSC in regaining tissue homeostasis and enhancing tissue regeneration. For example, exosomes derived from BM-MSCs (Collino et al., 2017) play a protective role in mice with acute renal injury. It involves the transfer of mRNAs and microRNAs to injured adrenocortical cells, which results in the reduction of apoptosis. Zhu et al. (2014) reported that MSC-derived exosomes alleviate acute lung injury (ALI) triggered by endotoxin in mice by transferring keratinocyte growth factor (KGF) mRNA to the injured alveoli. Previous studies also demonstrated that owning to the ability to cross the blood-brain barrier, MSC-derived exosomes may become an ideal therapy option for various degenerative brain disorders such as Parkinson’s disease, stroke, autism and Alzheimer’s disease (Perets et al., 2019; d'Angelo et al., 2020; Saint-Pol et al., 2020). Furthermore, modified exosomes were shown to be an effective vehicle for the administration of physiologically active components as well as medications. In addition to protecting payloads from enzymatic destruction, exosomes also enable them to target cells in a passive and particular manner. Overall, this is achieved due to their distinctive double-layered membrane structure (Liang et al., 2020; Han et al., 2021).

Moreover, in musculoskeletal repair and regeneration, the research using exosomes is still in its infancy. In previous studies (Pourakbari et al., 2019; Mohd Noor et al., 2021), the chondroprotective, anti-inflammatory, and antioxidant characteristics of MSC-derived exosomes have been found to deliver promising therapeutic benefits in the treatment of osteoarthritis (OA). As the most common forms of degenerative joint diseases, the phenotypic signs of OA and IVDD are very similar despite some obvious differences in their etiology and pathophysiology (Rustenburg et al., 2018). This suggests that at least a part of the treatment mechanism using MSC exosomes in OA can be used for the repair of intervertebral disc degeneration.

## 8 Impacts of MSC-Derived Exosomes on IVDD

Although the application of MSC-derived exosomes in IVDD treatment is still at an early stage, it is clear that there is growing interest in its use. Reduced ECM content, cell loss, inflammation, and increased oxidative stress are the pathophysiological hallmarks of IVDD. Encouragingly MSC-derived exosomes may restore the deteriorated IVD by targeting these four elements of the disease. Table 2 summarizes the findings of existing studies using MSC-derived exosomes in IVD regeneration and repair. In these 16 rescue-based research, MSC-derived exosomes were extracted from a number of cell sources, such as placenta-MSCs (1 study), umbilical cord (UC)-MSCs (1 study), adipose (AD)-MSCs (1 study), BM-MSCs (10 studies), unspecified (2 studies) tissues and human-induced pluripotent stem cells (1 study). Despite the differences in the tissue source, MSC-derived exosomes exert similar therapeutic effects in a pre-clinical model of IVDD mainly by restoring ECM homeostasis, promoting cell proliferation, reducing cell death, regulating inflammatory response, and attenuating oxidative stress. The specific mechanisms are described below:

**TABLE 2:** Pre-clinical studies investigating the therapeutic potential of stem cell-derived exosomes on IVDD.

Study	Cell type	Species of cell source	Size of EV	Isolation	Model	Modeling method	*In vitro* appraisement	*In vixo* appraisement	Additional Manipulation
Qi et al.	UC-MSCs	Human	**—**	Ultra-centrifugation	*In vitro*	**—**	1. MSC-exosome has the potential to alleviate HG induced extracellular matrix degradation via the p38 MAPK pathway	**—**	High glucose (HG) induced degradation of IVD
							2. HG also significantly inhibited collagen II and aggrecan expression in NPMSCs, and leads to an increase in the rate of NPMSCs apoptosis		
							3. miRNAs array results and bioinformatics analysis predicted that miR-221-3p, let-7a-5p, and miR-21-5p derived from hUCMSCs-EXO may be closely related to extracellular matrix synthesis of NPMSCs		
Li et al.	BM-MSCs	Human	around 100 nm	Ultra-centrifugation	*In vitro*	**—**	1. In the pathological acid environment, MSC-derived exosome promotes the expression of chondrocyte extracellular matrix, collagen II, and aggrecan, and reduces matrix degradation by downregulating matrix-degrading enzymes.	**—**	Acidic pH-induced NPCs apoptosis
							2. MSC-derived exosomes was able to prevent and mitigate NPC apoptosis through repressing caspase---		
							3 expression and attenuating caspase-3 cleavage induced by acidic pH		
Li et al.	BM-MSCs	Human	around 100 nm	Ultra-centrifugation	*In vitro*	**—**	1.BM-MSC-derived exosomes inhibits IL-1b-induced inflammation and apoptosis of AF cells by suppressing PI3K/AKT/mTOR signaling pathway-mediated autophagy	**—**	IL1-β treatment (10 ng ml^−1^)
							2. BM-MSC-exosomes supported AF cell viability after IL1-β treatment		
Yuan et al.	PLMSCs	Human	30–150 nm	Ultra-centrifugation	*In vitro In vivo*	**—**	1. MSC exosomes riched in antagomiR-4450 ameliorates NPC damage by promoting proliferation and migration	1.The EXO-antagomiR-4450 attenuated IVDD damage by repressing miR-4450 and upregulating ZNF121 expression	TNF-α (10 ng ml^−1^) treatment
							2.EV-derived AntagomiR-4450 decreased MMP13, IL6, IL1-β, CASP3 expression, and increased COL2 and ACAN expression	2.The EXO-antagomiR-4450 could partially decline the pro-inflammatory factors and MMPs in IDD mice	
							3.miR-4450 was significantly upregulated while ZNF121 was downregulated in IDD and miR-4450 exacerbated NPC damage by targeting ZNF121		
Zhu et al.	BM-MSCs	Rat	mainly 109.3 nm	Ultra-centrifugation	*In vitro*	**—**	1. MSC-exosomes could inhibit TNF-α-induced increase of apoptotic process, activation of apoptotic proteins, imbalance of anabolism/catabolism levels, and accumulation of collagen I in NPc through the delivery of miR-532–5p	**—**	TNF-α (20 ng ml^−1^) treatment
							2. RASSF5 is a direct target gene of miR-532–5p to mediate the cell apoptosis regulation		
Wen et al.	BM-MSCs	Rat	about 80 nm	Ultra-centrifugation	*In vitro In vivo*	Injection of absolute ethanol into sub-endplate in rat tail to induce IVD degeneration	1. The expression of col II and Aggrecan, SA-β gal positive cells and apoptosis rate of NPCs were decreased after MSC exosomes intervention	1.After MSCs-exosomes treatment, MMP-2, MMP-6, TIMP1 and TUNEL-positive cells were decreased, and miR-199a was increased in IDD mice	**—**
							2. Reducing miR-199a carried by MSC exosomes led to an impaired protective effect of exosomes on NPCs	2. BMSCs-EVs promote proliferation of NPCs and inhibit apoptosis	
							3. miR-199a from MSC exosomes promotes repair by targeting GREM1 and downregulating the TGF-β pathway		
Zhu et al.	BM-MSCs	Mouse	80 nm	Ultra-centrifugation	*In vitro*	**—**	1.MSCs exosomes alleviated NPCs apoptosis by reducing IL-1β-induced inflammatory cytokines secretion and MAPK signaling activation	**—**	IL1-β treatment (10 ng ml^−1^)
							2. MSCs exosomes inhibited NPCs apoptosis and MAPK signaling by delivering miR-142-3p that targets mixed lineage kinase 3 (MLK3)		
Cheng et al.	BM-MSCs	Human	average 87 nm	Ultra-centrifugation	*In vitro In vivo*	The model of disc degeneration was established by needle puncture. (needle-stab model)	1. MSC-exosomes were taken up by NPCs and suppressed NPC apoptosis induced by TNF-a	1. Intradiscal injection of MSC-exosomes alleviated the NPC apoptosis and IVD degeneration in a rat model	TNF-α (5 ng ml^−1^) treatment
							2.miR-21 in MSC-exosomes alleviated TNF-a induced NPC apoptosis		
							3.Delivery of miR-21 in MSC-exosomes inhibited NPC apoptosis by targeting PTEN through PI3K-Akt pathway		
Zhang et al.	MSCs	Human	around 100 nm	Ultra-centrifugation	*In vitro In vivo*	The IVDD model was established by annulus fibrosus (AF) needle puncture	1) MSCs-derived exosomes play an anti-pyroptosis role by suppressing the NLRP3 pathway	1. MSCs-exosomes and miR-410 treatment alleviated the severity degree of IVDD	LPS (5 mmol L^−1^) treatment
							2) MSC-derives exosomes treatment inhibit LPS-induced pyroptosis in NPCs		
							3) miR-410 derived from MSC exosomes inhibit LPS-induced pyroptosis in NPCs		
Hingert et al.	BM-MSCs	Human	average 144 ± 2.22 nm	Serial centrifugation	*In vitro*	**/**	1.MSC exosomes treatment increased cell viability and proliferation of degenerated disc cells	**/**	No
							2. MSC exosomes treatment induced early production of crucial ECM components such as proteoglycan, aggrecan, and collagen type II		
							3. EV treatment suppressed apoptosis and the secretion of MMP-1 in disc cells		
Sun et al.	iMSCs	Rat	80–200 nm	Ultra-centrifugation	*In vitro In vivo*	The IVDD model was established by annulus fibrosus (AF) needle puncture	1. miR-140-5p riched in iMSC-exosomes played a pivotal role in the iMSC-sEV-mediated therapeutic effect by upregulating of anabolism markers of the ECM (collagen II and aggrecan), and downregulating of catabolism markers of the ECM (MMP-3 and ADAMTS-4)	1. NPC senescence and IVDD were significantly improved after intradiscally injecting iMSC-exosomes	No
							2. iMSC-sEVs could rejuvenate senescent NPCs and restore the age-related function by activating the Sirt6 pathway *in vitro*		
Lu et al.	BM-MSC	Human	30–100 nm	Ultra-centrifugation	*In vitro*	**—**	1) EV treatment increased proliferation activity of NPC	**—**	No
							2) EV treatment generate a healthier extracellular matrix by upregulating expression levels of anabolic/matrix protective genes (aggrecan, colla-gen II, sox-9 and TIMP-1) and downregulating matrix degrading genes (MMP1 and MMP3)		
Xia et al.	BM-MSCs	Mouse	50–130 nm	Ultra-centrifugation	*In vitro In vivo*	An IVD degeneration rabbit model was induced with a fine needle puncture	1. BMSC-derived exosomes attenuate apoptosis in NP cells treated with H_2_O_2_	1. Exosomes attenuate the progression of IVDD	H_2_O_2_(500 × 10^−6^_M_)
							2.Exosomes dampen H_2_O_2_-induced inflammatory marker expression and matrix degradation in NP cells	2. Exosomes delay matrix degradation during the progression of IVDD	
							3. Exosome attenuate TXNIP–NLRP3 inflammasome activation and caspase-1 cleavage induced by H_2_O_2_		
							4. Exosomes replenish mitochondrial-related proteins and attenuate mitochondrial dysfunction		
Bari et al.	ASCs	Human	171.8 ± 18.3 nm	Ultrafiltration	*In vitro*	**—**	1. at concentrations between 5 and 50 mg/ml, freeze-dried secretome showed to *in vitro* counteract the oxidative stress damage induced by H_2_O_2_ on nucleus pulposus cells	**—**	H_2_O_2_(1 × 10^−3^_M_)
							2. Freeze-dried secretome became cytotoxic to NPCs at a concentration of over 50 mg ml−1		
Xie et al.	MSCs	Rat	30–200 nm	Ultra-centrifugation	*In vitro In vivo*	The IVDD SD rat model was established by needle puncture	1. Exosomes reduced apoptosis and calcification of EPCs induced by TBHP	1.Sub-endplate injection of MSC-exosomes can ameliorate IVDD	TBHP (20 × 10−^6^ to 60 × 10^−6^m)
							2.The downregulated level of miR-31-5p in exosomes impaired exosomal protective effects on EPC.	2. Downregulation of miR-31-5p from exosomes inhibited exosomal protective effects on EPC	
							3. miR-31-5p negatively regulated ATF6-related ER stress and inhibited apoptosis and calcification in EPCs		
Liao et al.	BM-MSCs	Human	average 94.3 nm	ExoQuick reagent	*In vitro In vivo*	Injection of AGEs into in SD rat tail to induce IVD degeneration	1) MSC-exos reduced AGEs-induced ER stress and ameliorated NP cells apoptosis	1. MSC-exos modulated ER stress-related apoptosis and retarded IDD progression in a rat tail model	AGEs (200 μg ml^−1^)
							2) MSC-exos inhibited the activation of UPR under AGEs-induced ER stress, and decreased CHOP expression		
							3) MSC-exos attenuate ER stress-induced apoptosis by activating AKT and ERK signaling in human NPCs		

MSC, mesenchymal stem cell; BM-MSC, bone marrow-derived mesenchymal stem cell; NPC, nucleus pulposus cell; AFC, annulus fibrosus cell; UC-MSC, umbilical cord-derived mesenchymal stem cell; EPCs,endplate chondrocytes; ASC. adipose-derived mesenchymal stromal cell; CEPC, cartilage endplate chondrocyte; PLMSC, placental mesenchymal stem cell; TBHP, tert-butyl hydroperoxide; ER, endoplasmic reticulum; UPR, unfolded protein response; CHOP, C/E homologous protein; AGEs, Advanced glycation end products.

### 8 1 Restoring Extracellular Matrix Homeostasis

One of the most significant characteristics of IVDD is the disruption of ECM homeostasis (Lu et al., 2019). Normally, anabolism and catabolism of ECM in IVD cells are in a dynamic balance, which is regulated by matrix catabolic factors (including matrix metalloproteinases (MMPs), matrix anabolic factors (including transforming growth factor (TGF)), and disintegrins and metalloprotease with thrombospondin motifs (ADAMTSs) (Chen et al., 2019). In addition, tissue inhibitors of metalloproteinases (TIMPs) can also regulate the activity of matrix-degrading enzymes (Chu et al., 2019). Earlier research reports have confirmed that when aging, injury, genetics or other exogenous stimuli disrupt the dynamic balance within the disc, certain pro-inflammatory cytokines including interleukin (IL)-1β and tumor necrosis factor (TNF)-a, may be secreted from IVD cells as well as infiltrating immune cells and lead to abnormal ECM remodeling via the mechanism of the up-modulation of catabolic enzymes (ADAMTSs and MMPs) and the downmodulation of ECM synthesis-related molecules (proteoglycan, aggrecan, and collagen type II) (Risbud and Shapiro, 2014) (Johnson et al., 2015). As a consequence, enhancing anabolism while simultaneously decreasing catabolism is critical for preserving the integrity of ECM and slowing the degeneration of intervertebral discs.

An accumulating amount of available data demonstrates that MSC-exosomes may effectively enhance the expression of extracellular matrix-related molecules and simultaneously inhibit their decomposition, thereby storing the ECM homeostasis (Bao and He, 2021; Mohd Noor et al., 2021). In previous studies, researchers used different *in vitro* cellular challenges (including IL-1β) (Zhu L. et al., 2020; Li Z.-q. et al., 2020), high glucose (Qi et al., 2019), LPS (Zhang J. et al., 2020), advanced glycation end products (AGEs) (Liao et al., 2019a), hydrogen peroxide (H_2_O_2_) (Bari et al., 2018; Xia et al., 2019), TNF-ɑ (Cheng et al., 2018; Zhu G. et al., 2020; Yuan et al., 2020), tert-butyl hydroperoxide (TBHP) (Xie et al., 2020), and acidic pH (Li et al., 2020a)) to simulate the degenerative microenvironment. For these biochemical challenges, exosomes produced from MSCs have been shown to have a protective impact on NPCs via the mechanism of increasing the expression of chondrocytes, aggrecan, and collagen II in the extracellular matrix and decreasing matrix disintegration via the mechanism of the down-modulation of matrix-degrading enzymes. Moreover, the effects of MSC-derived exosomes on ECM metabolism in IVD cells without any biochemical challenge have also been investigated (Lu et al., 2017; Hingert et al., 2020). One study (Lu et al., 2017) showed that after the intervention of exosomes derived from MSC, the ECM expression in degenerated nucleus pulposus cells increases significantly. The up-modulation of TIMP-1 and downmodulation of MMP-1/MMP-3 also indicate a healthier balance between catabolism and anabolism. Similar findings were presented by Hingert et al. (2020) When comparing degenerated disc cell pellets treated with MSC-derived exosomes to control cultures, it was observed that ECM synthesis was over three times greater in the treated pellets. MMP-1 secretion in the degenerated disc cells was similarly reduced as a result of the exosome therapy.

Furthermore, subsequent research has proven the strong relationship between miRNAs and the progression of IDD, which partially explains the effective role and its underlying mechanism of MSC exosomes in the IVD (Zhu G. et al., 2020; Wen et al., 2021). MicroRNA-199a in BMSC-secreted exosomes showed a superior protective role against ECM degradation by targeting GREM1 to inactivate the TGF-βpathway. Exosomes from BMSCs contain miR-532-5p, which inhibits matrix disintegration via the mechanism of targeting MMP-13 in degraded NPCs, resulting in the mitigation of IDD (Zhu G. et al., 2020). Moreover, Sun et al. demonstrated that iMSC-exosomes loaded exogenous miR-105-5p performed an integral function in the iMSC-sEV-mediated treatment impact by downregulating the catabolism markers of the ECM (ADAMTS-4 and MMP-3) and upregulating the anabolism markers (aggrecan and collagen II) (Sun Y. et al., 2021). This indicates that delivering exogenous micro-RNA via MSC exosomes might be a promising therapeutic approach for IVDD.

Together, the abovementioned findings suggest that MSC-derived exosomes have a pro-regenerative effect on terminally differentiated cells that have been derived from the degenerated IVD tissue via inhibition of catabolism and promotion of ECM synthesis.

### 8.2 Cell Proliferation Stimulation and Apoptosis Inhibition

In addition to an inflammatory environment, the central portion of degenerating discs comprises hypoxia, low glucose, elevated osmotic pressure, increased mechanical loading, low pH, and nutrient deficiency, which decreased cell viability and increased cell apoptosis (Ding et al., 2013; Sakai and Andersson, 2015). Exosomes produced from MSCs have been shown to enhance the proliferation of IVD cells while simultaneously inhibiting their apoptosis (Hu et al., 2020).

#### 8.2.1 NP Cells

Most of the studies regarding IVD degeneration have been focused on nucleus pulposus cells (NPCs), which are the resident cells of the disc and perform instrumental functions in maintaining the normal function of IVD (Jia et al., 2016). Excessive nucleus pulposus cells apoptosis is one of the main reasons for the initiation and progression of IVDD (Guo et al., 2021). Previous studies showed that the co-culture of MSCs with nucleus pulposus cells not only stimulated the NPCs proliferation but also attenuated cell apoptosis in the degenerated IVD. Studies have shown that exosomes derived from MSC replicate these therapeutic effects of their parent cells (Faruqu et al., 2021; Zhu et al., 2021). For *in vitro* disc degeneration model induced by different biochemical challenges, including H_2_O_2_, LPS, IL-1β, TNF-ɑ, acidic pH, and high glucose, the administration of MSC-derived exosomes exerts pro-proliferation and anti-apoptosis effect via distinct mechanisms. One study (Cheng et al., 2018) reported that MSC-derived exosomes promoted NPC proliferation and prevented apoptosis in NPCs, and alleviate IVD degeneration through miR-21. Exosomal miR-21 restrains PTEN activity and thus regulates PI3K/Akt pathway in NPC apoptosis. Another study (Li et al., 2020a) demonstrated that MSC-derived exosomes were capable of preventing and attenuating NPC apoptosis by suppressing caspase-3 expression and alleviating caspase-3 cleavage triggered by acidic pH. In addition, Zhu G. et al. (2020) discovered that exosomes originating from BMSCs inhibit fibrotic accumulation, ECM disintegration, NPC apoptosis, and alleviate the progress of IDD through miR-532–5p. The RASSF5 has been shown to directly target the miR-532–5p gene which mediates the regulation of cell apoptosis. Similarly, miR-142-3p from MSC exosomes also stimulate NPCs proliferation and ameliorate the apoptosis of the NPC induced by IL1-β *via* targeting MLK3 to suppress MAPK signaling (Zhu L. et al., 2020).

Yuan et al. (2020) found that overexpression of miRNA-4450 in nucleus pulposus cells could promote cell apoptosis and inflammatory responses; conversely, the above results were opposite upon knocking out miRNA-4450 in the cells. Further bioinformatic analysis showed that miRNA-4450 could specifically bind to the zinc finger protein 121. When co-cultured with MSC-derived exosomes, the apoptosis rate in nucleus pulposus cells treated with TNF-ɑ decreased significantly. Thus, MSC-derived exosomes may reduce the apoptosis in nucleus pulposus cells by antagonizing the signaling pathways involving miRNA-4450 and zinc finger protein 121. Wen et al. (2021) also report that miR-199a from MSC exosomes promote the proliferation of NPCs and inhibit cell apoptosis *via* downregulating GREM1. Moreover, a recent study by our team also testified that exosomes originating from MSC might alleviate apoptosis induced by advanced glycation end products (AGEs) *in vitro* via activating AKT and ERK signaling pathways (Liao et al., 2019a).

#### 8.2.2 Annulus Fibrosus Cells

Annulus fibrosus is a fibrous cartilage-like structure arranged in concentric circles outside the nucleus pulposus (van den Akker et al., 2016). It protects the jelly-like nucleus pulposus tissue and evenly distributes the pressure on it. Thus, the annulus fibrosus performs a critical function in maintaining the normal physiological function of the IVD. Li Z.-q. et al. (2020) found that BMSC-derived exosomes could not only promote proliferation but also significantly reduce the apoptosis induced by IL-1B in annulus fibrosus cells. Further studies show that IL-1B promotes autophagy-related proteins expression (LC3-II, LC3-I, and Beclin-1) and reduces the expression of P13K/mTOR/AKT signaling pathway-related proteins (p-mTOR and p-AKT). After the intervention using BMSC-derived exosomes, the effect of IL-1B could be reversed, and with the addition of rapamycin, an inhibitor of the H1TOR pathway, the expression of inflammatory cytokines and phagocytic associated proteins in BMSC-derived exosomes were significantly enhanced. Therefore, BMSC-derived exosomes may reduce the apoptosis in annulus fibrosus cells by inhibiting P13K/AKT/mTOR signaling pathway-mediated autophagy. Although there are few studies on the relationship between MSC-exosomes and annulus fibrosus apoptosis, given the important role of annulus fibrosus in IVDD, the inhibition of annulus fibrosus apoptosis by BMSC-derived exosomes need to be investigated in the future.

#### 8.2.3 Cartilage Endplate Cells

The cartilage endplate (CEP) is known to be a hyaline cartilage layer existing between the vertebral bodies and intervertebral disc. Due to their rich micropore structure that allows nutrients and metabolites to pass through, the CEP is the predominant source of nutrients for the avascular IVD (Raj, 2008). Degeneration of the CEP, including calcification and excessive apoptosis of CEP cells, could impede nutrient diffusion, resulting in metabolic and functional disorders of IVD cells to initiate IVDD (Sakai and Grad, 2015). Therefore, CEP degeneration may be a pivotal initiating factor in IVDD pathogenesis, and inhibition of early CEP degeneration is very important to delay IVDD.

Currently, only a study focus on the relationship between MSC-derived exosomes and apoptosis in cartilage endplate cells (Xie et al., 2020). MSC-derived exosomes were shown to be effective in preventing and inhibiting apoptosis as well as calcification of cartilage endplate cells once exposed to oxidative stress caused by tert-butyl hydroperoxide (TBHP). Further analysis showed that MSC exosomes are rich in miRNA-31-5p, whereas down-modulation of miR-31-5p inhibited exosomal protective benefits on CEP. Pathway analysis showed that ATF6 was the miR-31-5p target, and the ATF6 expression was inversely modulated by miR-31-5p. It has also been shown thatATF6 can trigger cell apoptosis via the ER-stress pathway. CEPs were less susceptible to cell death and calcification when treated with MSC-exosomes, and the specific mechanism might be correlated with the pathway modulation of the miR-31-5p/ATF6-associated ER stress.

### 8.3 Anti-Oxidation Effects

Excessive oxidative stress is key in the early events of IVDD (Luo et al., 2019). It promotes the expression of catabolic factors, including MMP-3 and TNF-α (Song et al., 2018). Previous research shows that the pathological mechanisms underlying IVDD are closely associated with ROS and oxidative stress (Feng et al., 2017). Throughout the course of NP cell degradation, the homeostasis between the production of oxygen-free radicals and antioxidant defense is interrupted, thereby resulting in the buildup of reactive oxygen species (ROS), oxidative stress, and ultimately mitochondrial apoptosis in NPCs. Thus, anti-oxidative stress mechanisms are a feasible pathway for IVDD treatment. Recently, Bari et al. reported that although the freeze-dried secretome shows no free-radical scavenging activity, it was shown to be effective in protecting oxidative damage caused by oxidative stress at concentrations ranging from 5 to 50 mg/ml. According to the researchers, this capacity was due to the induction as well as the regeneration of physiologically active antioxidant systems inside cells (Bari et al., 2018). Xia et al. showed that attenuation of mitochondrial dysfunction caused by oxidative stress may greatly improve cellular homeostasis in NP cells once expose to IVDD. They hypothesized that MSC exosomes may provide mitochondrial proteins to NP cells, enabling the cells to repair the damaged mitochondrial components. The proteomic database developed by them also indicates that MSC exosomes contain antioxidant proteins including Prdx-1, Gpx 4, and Trap 1 (Xia et al., 2019). The separation and accurate identification of these proteins, nevertheless, would need more research. Moreover, our prior research showed that AGEs accelerated the onset of IDD by exacerbating mitochondrial dysfunction as well as oxidative stress. Inspired by this work and the therapeutic potential of MSC exosomes, we carried out a series of tests and demonstrated that MSC-derived exosomes might attenuate AGE-induced ER stress *in vitro* via the activation of the ERK and AKT signaling pathways. In addition, an *in vivo* trial demonstrated that the administration of MSC exosomes had a therapeutic impact on IVDD (Liao et al., 2019a).

### 8.4 Anti-Inflammatory Effects

Numerous studies have demonstrated that disc degeneration is associated with inflammatory response (Yang et al., 2015). During IVDD, degenerative intervertebral disc cells themselves produce proinflammatory chemokines includingCCL545, CCL4, CCL3, CXCL1, and XCL1 that are able to attract other leukocytes and produce pro-inflammatory cytokines including TNF-ɑ, IL-1, IL-2, IL-6, and IL-8 that quicken the IVDD progression by upmodulating the matrix catabolic enzymes (Cunha et al., 2018). As the degeneration cascade progresses, the production of pro-inflammatory molecules, particularly TNF–αand IL-1, increases rapidly. Although similar therapeutic effects were observed in these studies, the mechanism of action underlying MSC exosome-related alleviation of inflammation corresponds to the differences in the biochemical challenge. Exosomes from MSCs have been shown to have an anti-inflammatory effect on pathogenic NP cells via the mechanism of inhibiting the production of inflammatory markers and the activation of the NLRP3 inflammasome (Xia et al., 2019). MSC-exosomal miR-410 targets the 3′UTR of NLRP3 and reduces NPC pyroptosis after LPS treatment (Zhang J. et al., 2020).

Moreover, the accumulation of inflammatory factors can also increase the apoptosis of the annulus fibrosus, destroy its structure and function, and further aggravate the process of IVDD. Li et al. reported that exosomes generated from BMSC could not only enhance the proliferation of annulus fibrosus cells and reduce the apoptosis induced by IL-1B but could also reduce the production of inflammatory factors including prostaglandin E2, IL-6, and cyclooxygenase-2. Further analysis showed that BMSC-derived exosomes could reduce the inflammatory response in annulus fibrosus cells by inhibiting P13K/AKll/mTOR signaling pathway-mediated autophagy (Li Z.-q. et al., 2020). Zhu and his colleagues performed further investigations, demonstrating that BMMSCs-derived exosomes-packaged miR-142-3p mitigate inflammatory damage in NPCs via the mechanism of inhibiting the MAPK signaling pathway by targeting MLK3 (Zhu L. et al., 2020).

Therefore, the anti-inflammatory effects of MSC exosomes can combine the miRNA and corresponding messenger RNA functions to inhibit the formation of inflammatory bodies, the activation of inflammatory factors, and the inhibition of cell autophagy. Thus, reducing the series of inflammatory reactions could potentially delay IVDD.

## 9 Challenges and Prospects

Exosome-based therapy is a novel option for the regeneration and repair of intervertebral discs. During the last decade, many research reports have revealed the therapeutic potential of MSC-derived exosomes in the treatment of a variety of illnesses. However, current investigations into the therapy of IVDD using exosomes derived from MSCs are at the stage of exploration; the majority of the preclinical trials have revealed potential benefits of MSC exosomes on NPCs; only one study reported the effects of MSC exosomes in AF cells; one study used CEP chondrocytes. For future investigations on therapeutic application, studies should focus on a comprehensive understanding of the regeneration and protection characteristics of this treatment approach on all terminally differentiated types of cells in IVD. Furthermore, the functions of exosomes produced from primordial cells in the human IVD in the regulation of regeneration and repair in IVDD should be explored further in the future. Furthermore, most preclinical studies on IVDD have used rodents, which compared to humans, have inherent differences between species (Lisieski et al., 2018). In addition, drug-induced or acupuncture-induced IVD degenerative animal models and *in vitro* cell line models may not reflect the true effect of exosomes on human intervertebral disc degeneration.

As a carrier of natural source signal molecules, exosomes have the advantages of nano-scale size, the permeability of biological barriers, low immunogenicity, low cytotoxicity, convenient storage, and their clinical applications are rapidly emerging (Hade et al., 2021). Exosomes generated from MSCs have gained widespread attention in the field of regenerative medicine due to their promising potential to regenerate damaged tissues on a par with MSCs themselves (Sakai and Grad, 2015). Furthermore, earlier research has proven that MSC-derived exosomes are appropriate alternatives to available MSCs for the purpose of overcoming the limits of these cells (Akao et al., 2011). However, although many preclinical studies demonstrated the good therapeutic potential of MSC-derived exosomes for IVDD, several critical issues will require careful consideration before using MSC exosomes in clinical settings. First, the absence of worldwide uniformity in the purification, separation, and profiling of MSC exosomes may result in controversial outcomes across various laboratory-based investigations. Uniform consensus on these aspects should be adopted to facilitate comparative research. Moreover, the preservation, transit, and storage of exosomes are crucial, which should be taken into consideration seriously. In spite of the fact that a study has shown that freeze-drying can purify exosomes and facilitate transportation and storage (Bari et al., 2018); however, further studies are needed to prove whether freeze-drying can change the characteristics of exosomes. Second, although some research reports have shown that exosomes generated from MSC are effective and safe in IVDD treatment, the pharmacological characteristics, such as bio-distribution, bioavailability, targeting, and pharmacokinetics, of exosomes *in vivo* remain unknown (Ailuno et al., 2020). Further studies directed towards these characteristics are needed, so as to elucidate their mechanism of action. Third, the optimal route of administration (local or systemic), administration dosing, interval, potency assays, and minimizing dose toxicity has not been effectively solved at present (Pittenger et al., 2019). Fourth, it is necessary to optimize the culture conditions and isolation methods for exosomes (Elabd et al., 2016; Lui, 2021). In order to produce safer and more effective functionalized exosomes on a large scale to cater to the needs of clinical research, the selection of appropriate culture conditions is critical to the development of exosome-based treatments for IVDD in the future. In addition, effective and scalable exosomes isolation methods from MSCs culture medium also should be in compliance with good manufacturing practices capable of reproducible purity and potency, which are essential for the exosomes yield (Forsberg et al., 2020). Last, the therapeutic outcomes of exosomes follow mainly from their intrinsic cargoes like RNA, DNA, lipids, and proteins. Despite the protein and RNA compositions of these exosomes have been quantitatively characterized, the specific mechanisms involved in the regeneration process need to be further elucidated. Moreover, current studies have mostly focused on regulators of miRNA sorting into exosomes, the effects of other exosomal components such as ncRNA, lncRNA, circRNA, tRNA, lipids, and proteins on the physiological functions of exosomes need to be further studied in the future.

## 10 Conclusion

In conclusion, exosomes generated from a variety of MSC sources have been shown to effectively exhibit anti-apoptotic, anti-oxidative stress, anti-inflammatory, and ECM homeostatic capabilities when delivered to IVDD patients in pre-clinical investigations. Hence, their therapeutic biological functions may provide a novel potential cell-free treatment for the regeneration and repair of IVD while minimizing the risks associated with cell therapy. Our knowledge about the biology and application of MSC-derived exosomes, however, is still incomplete. Further studies on the optimal route, interval, and dose of administration, the underlying mechanisms of MSC exosomes biological action are essential for optimizing the therapeutic effects. Clinical trials of standardized and reproducible MSC-derived exosomes preparation are needed before they can be applied to clinical practices.
